# Clinical features of atopic dermatitis in pediatric patients with skin of color and comparison with different phototypes

**DOI:** 10.1111/srt.13614

**Published:** 2024-02-13

**Authors:** Astrid Herzum, Corrado Occella, Lodovica Gariazzo, Giulia Ciccarese, Carlotta Pastorino, Simona Matarese, Lucia Marasini, Gianmaria Viglizzo

**Affiliations:** ^1^ Dermatology Department IRCCS Istituto Giannina Gaslini Genova Italy; ^2^ Department of Dermatology Univeristà degli Studi di Foggia Foggia Italy; ^3^ Department of Neuroscience Rehabilitation Ophthalmology Genetics Maternal and Child Health University of Genoa Genova Italy

Dear Editor,

Atopic dermatitis (AD) is a common inflammatory disease of the skin with lifetime prevalence of 20% and with growing incidence, affecting not only industrialized countries but also low‐income countries.^1^ AD has its onset usually in early childhood, with a prevalence of around 15%–20% in children. Yet, its prevalence varies widely among geographic regions, reaching its highest peak (35%) among Swedish children and dropping to its lowest (0.65%) among Tunisian children.^2^

AD represents a significant burden on the patients’ quality of life (QoL) and on healthcare resources, placing patients also at increased risk of developing allergic conditions such as rhinitis and asthma.^1^, ^2^ Moreover, the weak skin barrier in AD facilitates the acquisition of infections, such as cutaneous and mucosal warts, and viral persistence, in addition to hindering therapeutic methods.^3^

Precocious recognition of AD is of utmost importance to avoid unnecessary medical procedures and to establish a successful treatment together with effective educational programs for pediatric patients and their caregivers. However, AD may have very different clinical features, and wide clinical heterogeneity exists across different skin types. Recently, the need to gain a deeper understanding of AD clinical features in the skin of color (SOC), is emerging, as AD features in SOC are too often underreported and pathologized, addressing clinical findings as “atypical.”^4^

In this study, we aimed to give AD a wider characterization among different shades of skin, by characterizing clinical signs of AD in SOC patients and by comparing these signs with those of non‐SOC patients. We conducted a retrospective study on all the consecutive AD patients attending the Dermatology Unit of the Pediatric Hospital IRCCS Giannina Gaslini in Genoa, from September to December 2023. All patients were visited for the first time. Genoa is a multicultural, multiethnic port city, that offers the possibility of studying AD in all skin types.

Patients were clinically investigated by two expert dermatologists (A.H. and C.O.), who collected and evaluated the following features: Fitzpatrick skin type, characteristic clinical signs of AD disease (site and type of the lesions, associated symptoms), chronic or remittent disease course, and personal or family history of atopy. Among the typical signs of AD, we assessed the presence of extensor eczema of the arms and legs, considered as “classic” in early childhood, and flexural eczema of the same regions, a major diagnostic criterion of AD in older children (aged > 24 months) to adults. Also, pityriasis alba, intense xerosis, nummular eczema, (consisting of papular elements on a xerotic erythematous background) and prurigo nodularis‐type lesions (consisting of hyperkeratotic, excoriated nodules) were evaluated. All patients were pediatrics (0−18 years), did not present other chronic dermatological diseases, and did not receive any systemic treatment.

The severity of AD was evaluated through the calculation of the eczema area and severity index (EASI).^5^ The severity of pruritus was assessed with the visual analogue scale (VAS) scale (0–10 score), while xerosis was assessed using the overall dry skin score (ODS), a scoring system evaluating the severity of dryness from “slight” xerosis, with faint scaling and roughness, to “moderate” xerosis, with a rough, whitish appearance; to “severe” xerosis, with uniform scales and definite roughness; to “extreme” xerosis, with advanced roughness, large scales, cracks, and redness.^6^ Fitzpatrick skin types IV–VI were considered SOC, while types I–III, non‐SOC. Chi‐square test, with Yates correction when needed, was used to assess the statistical association between the two groups of patients (non‐SOC and SOC).

Overall, 128 patients were included in the study, with a mean age of 6.1 years. Of the patients, 53 (41%) had SOC (31 male, 22 female, mean age 6.9 years), 75 (59%) non‐SOC (34 male, 41 female, mean age 5.7 years). The mean EASI score was 19.72 (22.0 SOC; 18.9 non‐SOC). All patients reported chronically relapsing disease and intense pruritus (VAS 10). Personal or family history of atopy was described in 43% of patients: 21% (11/53) of SOC, 59% (44/75) of non‐SOC patients. Severe and extreme xerosis was observed in only 27% of patients: mainly in SOC (40%; 21/53), while of non‐SOC patients, only 19% (14/75) had this grade of xerosis. The association between xerosis and SOC was statistically significant (*p* = 0.015575).

Flexural eczema was noted in the majority (66%) of cases: 53% (28/53) of SOC patients, 76% (57/75) of non‐SOC patients. It was statistically associated with non‐SOC patients (*p* = 0.006263). Extensor surface eczema was observed in 37% of cases: 45% (29/53) of SOC, 11% (8/75) of non‐SOC patients, indicating a statistical association between extensor eczema and SOC patients (*p* < 0.0001) (Figure 1). Nummular eczema was detected in 13% of cases: 21% (11/53) of SOC patients, 8% (6/75) of non‐SOC. The association was statistically significant (*p* < 0.0001) (Figure 2). Prurigo nodularis‐type lesions were observed in 16% of patients: 32% (17/53) of SOC patients, 5% (4/75) of non‐SOC patients. A statistically significant association with SOC was highlighted (*p* = 0.000156) (Figure 1). Pityriasis alba was recorded in 28% of patients: 47% (25/53) of SOC patients, 15% (11/75) of non‐SOC patients.

**FIGURE 1:**
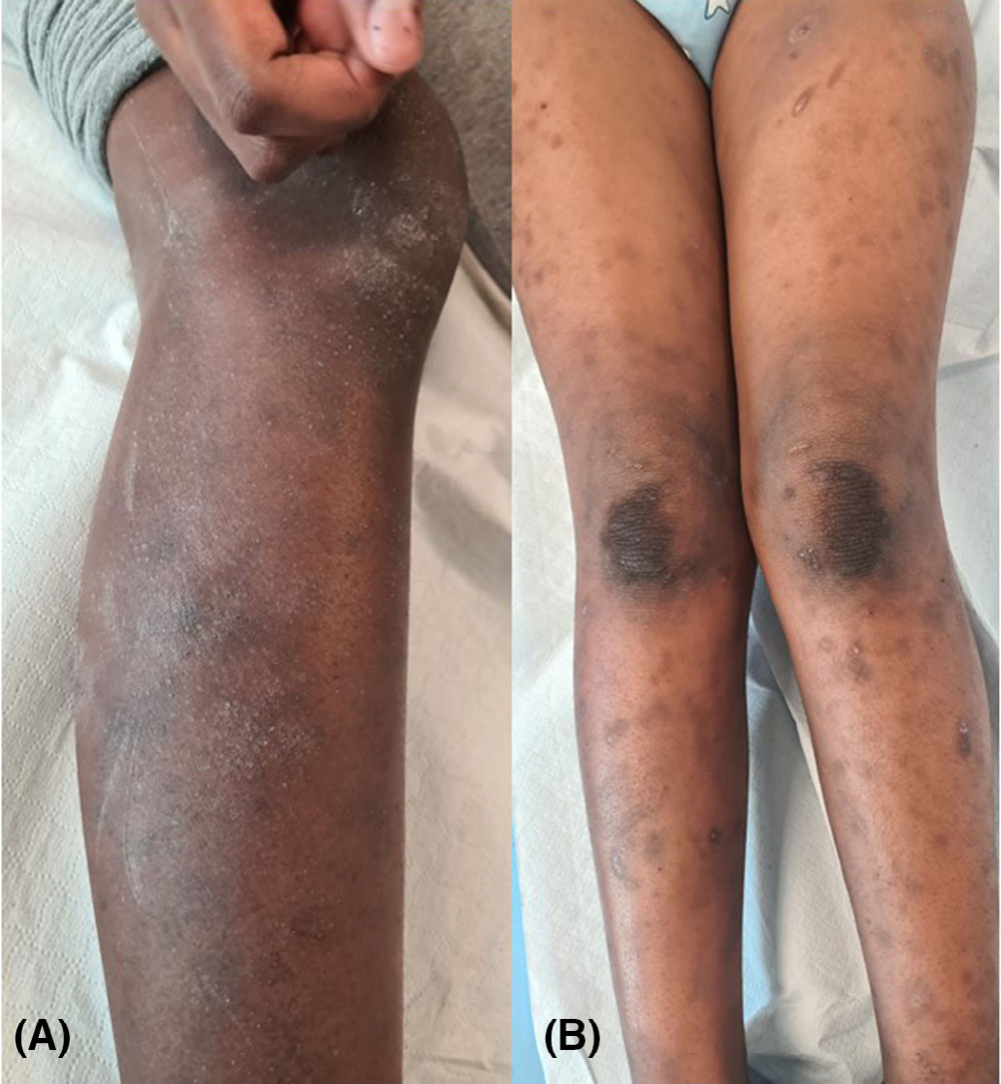
Intense xerosis of the legs associated with extensor surface eczema (A, B) and with prurigo nodules (B).

**FIGURE 2:**
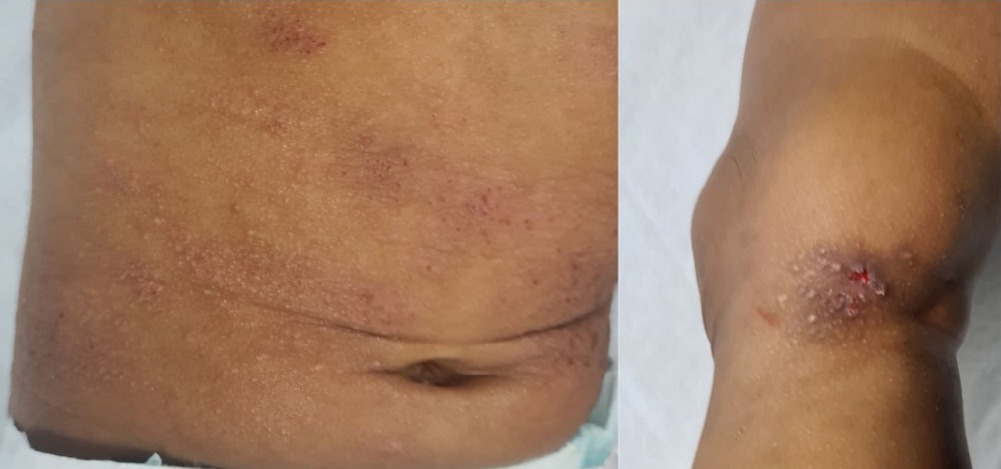
Nummular eczema, composed of papular elements on a xerotic erythematous background, of the trunk and legs.

From the present study, “typical” flexural distribution of the eczematous lesions emerged to be characteristic of non‐SOC (45%), while the most prevalent distribution of eczema in SOC was on extensor surfaces of the arms and legs (74%). A “typical” sign of AD in school‐aged children is flexural eczema of the arms and legs, representing a major diagnostic criterion of AD. As expected, the studied population of 6.1 years mean age, showed flexural eczema in the majority (66%) of cases. In line with the literature, the “typical” flexural eczema was more frequently observed in non‐SOC patients (*p* = 0.006263).^4^ This may be attributed to the difficulty of detecting erythema in SOC. Indeed, SOC skin inflammation appears as violaceous or darker brown, resulting in a lower color contrast with background skin compared to non‐SOC patients.^7^ The same issue of contrasts should arise when evaluating erythema in every other SOC body area. However, extensor surface eczema was observed in 45% of SOC, and only in 11% of non‐SOC patients, demonstrating a statistical association between extensor eczema and AD in SOC patients (*p* < 0.0001).

Nummular eczema had an overall low prevalence (13%), in line with other literature data, reporting a prevalence of 15%–17% in AD and the occurrence of eczematous lesions typically on extensor surfaces and on the trunk.^4^ Nummular eczema, prurigo nodularis, and intense xerosis, were observed mainly in SOC patients (respectively 21%, 32%, and 40%) (*p* < 0.001). As suggested by Muizzuddin et al., SOC is more likely to develop an intense level of xerosis, as a result of higher protein cohesion and lower ceramide levels in the upper layers of the stratum corneum.^8^ Conversely, filaggrin gene mutations seem to be of lower importance in SOC, while it is known that they represent a major risk factor for developing skin barrier dysfunction in non‐SOC.^9^, ^10^

Overall, we found that the main clinical features of AD in SOC were intense xerosis and xerosis‐related features such as nummular eczema, prurigo nodularis‐type lesions, and extensor site eczema. Indeed, the typical SOC‐related eczema involves the extensor surfaces of the arms and legs, known for being dry and exposed to irritant external agents, with features of hyperkeratosis (e.g., prurigo nodules) and typical nummular shapes.

As the evidence on the interracial genetic variability of AD is growing,^10^ it becomes clear that also the clinical features of AD are wider than previously described in only light‐skinned focused dermatology. These features deserve to be adequately described and addressed, as they might indeed represent underestimated major issues in SOC patients and might help with a more complete digital assessment of atopic dermatitis with teledermatology.^11^

## AUTHOR CONTRIBUTIONS

**Astrid Herzum; Lodovica Gariazzo; Simona Matarese; and Giulia Ciccarese** gave substantial contribution to conception and design; acquisition of data; interpretation of data; drafted the article and revised it critically; and gave final approval of the version to be published. **Corrado Occella** and **Gianmaria Viglizzo** gave substantial contribution to conception and design; acquisition of data; interpretation of data; revised the article critically; and gave final approval of the version to be published. **Carlotta Pastorino** gave substantial contribution to acquisition of data and gave final approval of the version to be published. **Lucia Marasini** gave substantial contribution to design; acquisition of data; interpretation of data; revised the article critically; and gave final approval of the version to be published.

## CONFLICT OF INTEREST STATEMENT

The authors declare no financial or non‐financial interests that are directly or indirectly related to the work submitted for publication.

## Data Availability

The data that support the findings of this study are available from the corresponding author upon reasonable request.
